# Posttraumatic stress disorder and risk of selected autoimmune diseases among US military personnel

**DOI:** 10.1186/s12888-020-2432-9

**Published:** 2020-01-15

**Authors:** Deborah Boggs Bookwalter, Kimberly A. Roenfeldt, Cynthia A. LeardMann, So Yeon Kong, Mark S. Riddle, Rudolph P. Rull

**Affiliations:** 1 Deployment Health Research Department, Naval Health Research Center, 140 Sylvester Rd., San Diego, CA 92106 USA; 2 Westat, 1009 Slater Rd. Suite 110, Durham, North Carolina 27703 USA; 3 Leidos, 11951 Freedom Dr., Reston, Virginia 20190 USA; 4 School of Medicine, University of Nevada, Reno, 1664 North Virginia Street, Reno, NV 89557 USA

**Keywords:** Posttraumatic stress disorder (PTSD), Autoimmune disease, Military, Cohort studies

## Abstract

**Background:**

Increasing evidence suggests a link between posttraumatic stress disorder (PTSD) and physical health. Stress disorders may lead to impairment of the immune system and subsequent autoimmune disease. This study investigated the association between PTSD and risk of selected autoimmune diseases (i.e. rheumatoid arthritis, systemic lupus erythematosus, inflammatory bowel diseases, and multiple sclerosis) among US active duty service members.

**Methods:**

Using data from the Millennium Cohort Study, incident autoimmune cases between study initiation and September 2015 were identified from medical encounter records in the Military Health System Data Repository (MDR). Participants were classified as having a history of PTSD if they self-reported receiving a health care provider’s diagnosis of PTSD or if they screened positive using the PTSD Checklist−Civilian Version. Hazard ratios (HRs) and 95% confidence intervals (CIs) were estimated using multivariable Cox regression models adjusted for demographics and history of another mental health condition.

**Results:**

Among 120,572 participants followed for a mean of 5.2 years, risk of any of the selected autoimmune diseases was 58% higher for those with a history of PTSD (HR = 1.58, 95% CI: 1.25, 2.01) compared with no history of PTSD. Further adjustment for BMI, smoking status, and alcohol use had little impact on the effect estimates, and results were not appreciably different according to combat experience and history of physical or sexual trauma.

**Conclusions:**

Active duty military personnel with PTSD may have an elevated risk of a range of autoimmune diseases, regardless of combat experience or prior trauma. Future research is needed to understand potential mechanisms which may inform future mitigative strategies in reducing extra-neuropsychiatric health problems among those with PTSD.

## Background

Posttraumatic stress disorder (PTSD) is a psychiatric disorder that can result from experiencing or witnessing a traumatic event, such as a serious accident or violent assault. Compared to the general population, military personnel are often exposed to dangerous situations and traumatic events, and therefore are at higher risk for mental disorders [1–3]. Some specific military experiences, such as combat and other deployment-related stressors, are associated with increased risk for PTSD [4]. In addition, sexual assault has been shown to increase risk for PTSD [5, 6] and the prevalence of sexual assault in the military exceeds that of the civilian population [7–9].

Individuals with PTSD remain in a perpetual state of hyperarousal and fear. As such, PTSD is associated with endocrine, immune, and neurobiological abnormalities. Posttraumatic stress has been shown to impede functioning of the hypothalamic pituitary adrenal axis, the autonomic nervous system, and the immune system [10–12]. Specifically, lower levels of glucocorticosteroids, elevated proinflammatory factors, altered gene expression, and accelerated aging of immune cells may lead to immune dysfunction that underlies the pathogenesis of multiple immune-mediated inflammatory diseases [10–16]. Furthermore, interactions within the neuroendocrine system are bidirectional [12], meaning that hyperarousal and subsequent increased hormonal activity in the central nervous system can trigger the release of excess levels of stress hormones that further contribute to chronic immune dysregulation and the potential development of autoimmune disease.

Findings from previous studies indicate that there is an association between PTSD and autoimmune diseases [10, 15–21]. A recent population- and sibling-matched retrospective cohort study of Swedish civilians with stress-related disorders found an elevated risk of autoimmune diseases, and those with PTSD had an additional elevated risk for multiple autoimmune syndromes, especially in younger individuals [15]. Another retrospective study of US veterans previously deployed to Iraq or Afghanistan who were receiving care from the Veterans Health Administration observed that veterans with an encounter for PTSD had elevated risks for rheumatoid arthritis (RA), systemic lupus erythematosus (SLE), multiple sclerosis (MS), inflammatory bowel disease (IBD), and thyroiditis compared with those seen for other psychiatric conditions as well as those without a psychiatric condition [16]. Female nurses enrolled in the Nurses’ Health Study II with probable PTSD had increased risk for developing RA and SLE [17, 18]. Elevated IBD risk was observed among US combat veterans with probable PTSD or anxiety/panic disorder [21] and among Danish civilians [20]. While these findings provide compelling evidence of an association between PTSD and autoimmune disorders, these studies were restricted to veterans after the end of their military service [10, 16, 19, 21], civilian populations [15, 17, 18, 20], or to a single gender [17–19]. Moreover, little is known about the potential role of combat and sexual assault in relation to PTSD and autoimmune disorders. Given the relatively young age at onset for many autoimmune diseases [22, 23] and the high prevalence of PTSD in the military, current military members comprise an important population in which to investigate risk of autoimmune disease in relation to PTSD and better understand the role of specific traumatic events.

Our study addresses this knowledge gap by using data from the Millennium Cohort Study, a large cohort of US service members. The primary objective of this study was to prospectively examine the association of PTSD and risk of several relatively common autoimmune diseases, including RA, SLE, MS, and IBD. The study population includes a large proportion of women, among whom many autoimmune diseases are most common [13, 24]. The secondary objective was to assess whether the association differed according to types of potentially traumatic experiences that are prevalent in a military population: history of combat exposure and history of physical or sexual trauma. Specifically, we hypothesized that (a) PTSD would be associated with an increased risk for RA, SLE, MS, and IBD and (b) that the magnitude of the association would be larger among individuals who experienced potentially traumatic experiences such as sexual trauma.

## Methods

### Study population

Launched in 2001, the Millennium Cohort Study is an ongoing prospective cohort study designed to investigate health effects associated with military service and has been previously described [25]. Briefly, four panels of US military personnel were enrolled in 2001, 2004, 2007, and 2011, leading to a total current enrollment of 201,619 from all service branches (Army, Navy, Marine Corps, Air Force, Coast Guard) and components (active duty, Reserve, National Guard). Enrolled participants are followed via self-administered surveys approximately every 3 years, regardless of military status at the time of follow-up. Baseline and follow-up surveys collected information on service-related experiences and behavioral, physical, and mental health. Data on demographic and military service-related characteristics were also obtained from electronic personnel files maintained by the Defense Manpower Data Center (DMDC). The study protocol was approved by the Naval Health Research Center Institutional Review Board (NHRC.2000.0007).

We initially restricted the analysis to 123,761 participants who were serving on active duty when they completed the baseline survey, due to the fact that active duty personnel have comprehensive access to the Military Health System during their time in service, while access to this system is limited for Reserve and National Guard personnel. We further excluded participants with evidence of autoimmune disease prior to baseline, either by self-report or from medical encounter data (*n* = 2221). After further excluding participants who were missing data on demographics, history of PTSD, or history of other mental health conditions (*n* = 968), the present analysis included 120,572 participants.

### Ascertainment of selected autoimmune diseases

The primary endpoints were RA, SLE, MS, IBD, and a composite outcome of any of the selected autoimmune diseases. Incident cases of selected autoimmune diseases were identified from medical encounter records in the Military Health System Data Repository (MDR) using the International Classification of Diseases, 9th Revision, Clinical Modification (ICD-9-CM) codes. Cases were defined as service members with one inpatient or at least two outpatient encounters with the corresponding ICD-9-CM code: 714 for RA; 710.0 for SLE; 340 MS; and 555 or 556 for IBD. The date of diagnosis was assigned as the earliest medical encounter noting the ICD-9-CM code of interest.

### Assessment of PTSD and covariates

PTSD and other mental health conditions were assessed at all available time points. Participants were classified as having a history of PTSD if they 1) self-reported receiving a health care provider’s diagnosis of PTSD, or 2) screened positive using the PTSD Checklist−Civilian Version (PCL-C). The PCL-C is a validated instrument used to rate the severity of 17 PTSD symptoms [26]. Using criteria established in the Diagnostic and Statistical Manual of Mental Disorders 4th edition (DSM-IV-TR), participants were classified as having a positive screen for PTSD if they reported a moderate or greater level of at least one intrusion symptom, three avoidance symptoms, and two hyperarousal symptoms [27].

Participants were classified as having a history of another mental health condition, with the exception of PTSD, if they 1) self-reported receiving a health care provider’s diagnosis of depression, schizophrenia, or bipolar disorder, or 2) screened positive for major depression, panic disorder, or other anxiety disorders using standardized Patient Health Questionnaire (PHQ) algorithms [28–30].

Age, sex, race/ethnicity, service branch, and pay grade were obtained from DMDC at the time of enrollment. Deployment in support of operations in Iraq and Afghanistan was determined based on dates in and out of theater from the Contingency Tracking System maintained by DMDC. Combat experience was defined as reporting at least one of the following: personally witnessing or being exposed to a person’s death due to war or disaster; witnessing physical abuse (torture, beating, rape); dead or decomposing bodies; maimed soldiers or civilians; or prisoners of war or refugees. Deployment dates in combination with the combat variable were used to assess if participants experienced no deployments, deployments without combat, or deployments with combat.

Data from all available study time points were used to ascertain height, weight, smoking status, alcohol intake, and prior physical/sexual trauma. Body mass index (BMI) was calculated as self-reported weight divided by the square of self-reported height (kilograms ÷ meters squared). Participants were classified as “never” smokers if they did not report that they had smoked at least 100 cigarettes (5 packs) in their lifetime; smokers were classified as former smokers if they reported having quit successfully or not having smoked in the past year. Heavy drinking was defined as exceeding recommended weekly limits of more than 14 and 7 alcoholic drinks per week for men and women, respectively. Alcohol misuse was measured as an affirmative response to any problem drinking item on the PHQ (e.g., “You drove a car after having several drinks or after drinking too much”) [28, 29]. Prior physical or sexual trauma was assessed based on any positive endorsement at baseline or follow-up to three items (e.g., “suffered forced sexual relations or sexual assault”, “experienced sexual harassment”, “suffered a violent assault”).

### Statistical analysis

Participants accrued follow-up time from the date of baseline survey completion to the date of diagnosis of the selected autoimmune disease (depending on the outcome of interest), separation from active duty status, or the end of the follow-up period (September 30, 2015), whichever occurred first. Multivariable hazard ratios (HR) and 95% confidence intervals (CI) were estimated with Cox proportional hazards models and adjusted for age, sex, enrollment panel, race/ethnicity (non-Hispanic white, non-Hispanic black, Hispanic, other), pay grade (enlisted or officer), service branch (Army, Navy, Marine Corps, Air Force), and history of another mental health condition. In sensitivity analyses, models were further adjusted for the following health behaviors: smoking status (never, former, or current), heavy or problem drinking (yes, no), and BMI (< 25, 25–29, ≥30 kg/m^2^). History of PTSD and other mental disorders, educational attainment, marital status, active duty status, military service branch, military rank, combat and deployment status, smoking status, BMI, alcohol intake, and prior physical/sexual trauma were updated as time-dependent variables in the analysis.

All analyses were stratified by sex due to known differences in autoimmune disease incidence between men and women [31]. Additional analyses were stratified by prior combat deployment experience and by prior physical or sexual trauma. All statistical analyses were performed using SAS version 9.4 (SAS Institute Inc., Cary, NC).

## Results

Age-standardized baseline characteristics by history of PTSD for the study population of 120,572 active duty U.S. military service members are listed in Table 1. Approximately 71% of the population was male while the baseline prevalence of PTSD was 8% among men and 9% among women. At baseline, both men and women with a history of PTSD were more likely than those without PTSD to be younger, of enlisted rank, in the Army, a current smoker, a heavy or problem drinker, or obese. Those with PTSD were also more likely to have a history of another mental health condition, combat experience, or physical or sexual trauma. Prior combat experience was more common among men, whereas prior physical or sexual trauma was more common among women (Table 1).

**Table 1:** Age-Standardized^a^ Baseline Characteristics (%) By History of PTSD, 2001–2015

Characteristic	All service members (*N* = 120,572)		Men (*N* = 85,460)		Women (*N* = 35,112)	
	No PTSD (*n* = 110,697)	PTSD (*n* = 9875)	No PTSD (*n* = 78,861)	PTSD (*n* = 6599)	No PTSD (*n* = 31,836)	PTSD (*n* = 3276)
Age, years^b^	27.6 (6.3)	26.2 (5.3)	28.1 (6.4)	26.5 (5.2)	26.4 (5.8)	25.7 (5.4)
Enrollment panel						
2001–2003	32	18	34	18	26	18
2004–2006	17	18	15	17	22	21
2007–2009	26	28	25	27	31	32
2011–2013	25	36	26	38	22	30
Race/ethnicity						
White, non-Hispanic	71	69	75	73	62	62
Black, non-Hispanic	13	12	10	10	20	18
Other	16	19	15	18	18	20
Officer rank	19	7	19	7	19	8
Service branch						
Army	34	53	34	55	34	49
Navy/Coast Guard	22	20	22	18	24	24
Marine Corps	10	13	13	16	5	6
Air Force	34	14	32	12	38	21
Other mental health condition^c^	8	67	5	63	13	74
Smoking status						
Never	60	42	58	39	66	49
Past	21	26	22	26	19	25
Current	19	32	21	35	15	27
Heavy or problem drinking	17	34	18	38	15	28
Body mass index^d^						
< 25	46	41	38	34	64	55
25–29	46	46	53	51	30	36
≥ 30	8	14	9	16	6	10
Prior combat experience	21	46	24	53	15	33
Prior physical or sexual trauma	10	31	6	20	19	53

^a^Values are standardized to the age distribution of the study population at the start of follow-up

^b^Values are expressed as mean (standard deviation)

^c^Participants were classified as having a history of another mental health condition if they self-reported a diagnosis of depression, schizophrenia, or bipolar disorder or if they screened positive for major depression, panic disorder, or other anxiety disorders

^d^Weight (kg)/height (m)^2^

During a mean follow-up of 5.2 years (SD, 3.7 years), 864 participants (55% men, 45% women) were identified with the autoimmune diseases of interest (309 RA, 103 SLE, 151 MS, and 332 IBD). Incidence rates for RA, SLE, and MS were higher for women than for men, whereas rates were similar for IBD (Table 2). For the composite outcome of any of the selected autoimmune diseases, risk was higher for those with a history of PTSD relative to those with no PTSD in a multivariable model adjusted for demographics and history of another mental health condition (HR = 1.6, 95% CI: 1.2, 2.0). The relative risk for a history of PTSD for each individual autoimmune disease ranged between 1.4 to 2.3 times that of those without a history of PTSD, though estimates were least precise for SLE and MS. Effect estimates stratified by sex did not differ appreciably except for MS (*P* for interaction = 0.007) and IBD (*P* for interaction = 0.12), but estimates were generally imprecise. Further adjustment for BMI, smoking status, and alcohol use did not appreciably change the magnitude of each effect estimate (results not shown in tables).

**Table 2:** Hazard Ratios for Selected Autoimmune Diseases By History of PTSD, 2001–2015

	All service members				Men				Women			
	Cases	IR^a^	HR^b^	95% CI	Cases	IR^a^	HR^b^	95% CI	Cases	IR^a^	HR^b^	95% CI
Any autoimmune disease												
No PTSD	755	1.3	1.0	Referent	417	1.0	1.0	Referent	338	2.2	1.0	Referent
PTSD	109	2.3	1.6	1.2, 2.0	56	1.7	1.4	0.9, 2.0	53	3.5	1.7	1.2, 2.4
Rheumatoid arthritis												
No PTSD	267	0.5	1.0	Referent	130	0.3	1.0	Referent	137	0.9	1.0	Referent
PTSD	42	0.9	1.4	0.9, 2.1	21	0.7	1.4	0.8, 2.5	21	1.4	1.4	0.8, 2.3
Systemic lupus erythematosus												
No PTSD	91	0.2	1.0	Referent	21	0.05	1.0	Referent	70	0.5	1.0	Referent
PTSD	12	0.3	1.4	0.7, 2.8	3	0.09	1.3	0.3, 6.0	9	0.6	1.4	0.6, 3.0
Multiple sclerosis												
No PTSD	127	0.2	1.0	Referent	56	0.1	1.0	Referent	71	0.5	1.0	Referent
PTSD	24	0.5	2.3	1.3, 3.9	16	0.5	3.5	1.7, 7.3	8	0.5	1.3	0.6, 3.0
Inflammatory bowel disease												
No PTSD	296	0.5	1.0	Referent	214	0.5	1.0	Referent	82	0.5	1.0	Referent
PTSD	36	0.8	1.6	1.0, 2.4	19	0.6	1.0	0.6, 1.7	17	1.1	3.2	1.7, 5.9

^a^Rate per 1000 person-years

^b^Adjusted for age, sex, enrollment panel, race/ethnicity, pay grade, service branch, and history of another mental health condition

Effect estimates from analyses stratified by combat deployment experience are listed in Table 3. Similarly, a history of PTSD was associated with elevated risks for the composite outcome among both those with no history of combat deployment (HR = 1.5, 95% CI: 1.1, 2.1) and those who had previously deployed and experienced combat (HR = 1.7, 95% CI: 1.2, 2.4) for men and women combined. Overall, these results did not significantly differ by sex (all *P* for interaction > 0.5).

**Table 3:** Hazard Ratios for Selected Autoimmune Disease By History of PTSD and Combat Deployment, 2001–2015

	All service members				Men				Women			
	Cases	IR^a^	HR^b^	95% CI	Cases	IR^a^	HR^b^	95% CI	Cases	IR^a^	HR^b^	95% CI
No combat deployment												
No PTSD	534	1.3	1.0	Referent	262	0.9	1.0	Referent	272	2.3	1.0	Referent
PTSD	48	2.3	1.5	1.1, 2.1	17	1.4	1.3	0.7, 2.2	31	3.6	1.6	1.1, 2.5
Combat deployment												
No PTSD	221	1.2	1.0	Referent	155	1.1	1.0	Referent	66	2.0	1.0	Referent
PTSD	61	2.3	1.7	1.2, 2.4	39	1.9	1.5	0.9, 2.3	22	3.4	2.0	1.1, 3.7

^a^Rate per 1000 person-years

^b^Adjusted for age, sex, enrollment panel, race/ethnicity, pay grade, service branch, and history of another mental health condition

In addition, effect estimates were not appreciably different according to history of physical or sexual trauma (Table 4). Overall, those with a history of PTSD had higher risk of any selected autoimmune disease among those with no prior trauma (HR = 1.5, 95% CI: 1.1, 2.0) as well as among those with a history of trauma (HR = 1.6, 95% CI: 1.1, 2.5); the findings did not appear to different between men and women (all *P* for interaction > 0.4).

**Table 4:** Hazard Ratios for Any Selected Autoimmune Disease According to History of PTSD and prior trauma, Millennium Cohort Study, 2001–2015

	All service members				Men				Women			
	Cases	IR^a^	HR^b^	95% CI	Cases	IR^a^	HR^b^	95% CI	Cases	IR^a^	HR^b^	95% CI
No prior trauma												
No PTSD	642	1.2	1.0	Referent	384	1.0	1.0	Referent	258	2.1	1.0	Referent
PTSD	68	2.0	1.5	1.1, 2.0	43	1.7	1.3	0.9, 1.8	25	3.2	1.7	1.1, 2.8
Prior trauma												
No PTSD	113	2.1	1.0	Referent	33	1.2	1.0	Referent	80	2.8	1.0	Referent
PTSD	41	3.0	1.6	1.1, 2.5	13	2.1	2.1	0.9, 4.5	28	3.8	1.5	0.9, 2.5

^a^ Rate per 1000 person-years

^b^ Adjusted for age, sex, enrollment panel, race/ethnicity, pay grade, service branch, and history of another mental health condition

## Discussion

In this large prospective study of active duty U.S. military service members, a history of PTSD was associated with an increased risk of selected autoimmune diseases during an average of 5 years of follow-up. Although estimates for each individual autoimmune disease were generally imprecise, there was an approximately 60% increased risk for the composite outcome of any of the selected autoimmune diseases —RA, SLE, MS, and IBD—for those with a history of PTSD relative to those without a history of PTSD. The magnitude of this association did not materially differ between men and women, though as expected absolute incidence rates were generally higher among women. In addition, findings did not differ according to specific types of prior trauma and healthy behaviors did not appear to influence this association between PTSD and autoimmune disorders.

Our findings from a longitudinal cohort of relatively young service members corroborate those from previous studies that examined the association between PTSD and autoimmune disorders among civilians and veterans. A recent retrospective cohort study of Swedish civilians reported that those with stress-related disorders as well as those with PTSD were more likely to develop an autoimmune disorder compared with those without a stress-related disorder [15]. However, this study was not able to adjust or examine the impacts of any lifestyle behaviors. In a large study of veterans enrolled in the Veterans Health Administration (VHA) who had deployed to Iraq or Afghanistan, PTSD was also associated with an increased risk of several autoimmune diseases considered individually [16], including each of those examined in the current study. The prevalence of PTSD in that study using electronic medical encounter data from the VHA was approximately 31% [16], compared with 8% in the present study of active duty personnel using a combination of a self-reported diagnosis or a positive screen using the PCL-C. Similar to our results, the observed increase in risk in the VHA study was evident among both those with and without a history of sexual trauma, as identified through clinical encounter data; however, the study did not have information on prior combat exposure [16].

A previous study using data from the first two enrollment panels of the Millennium Cohort reported a higher risk of IBD for an increasing number of life stressors; in addition, there was some evidence for elevated IBD risk among those with PTSD, though effect estimates were imprecise due to a small number of cases with PTSD [32]. Findings from this current study provide additional evidence that those with a history of PTSD have elevated risk for IBD, however, a significant risk was only detected among women. Further examination of IBD subtypes, including Crohn’s disease and ulcerative colitis, confirmed that point estimates were similar to the overall IBD risk, yet effect estimates were imprecise due to small case numbers (results not shown in tables). Additional research examining differential effects of PTSD by IBD subtype and among women is needed. Other prior work in the cohort suggests individuals who experienced combat while deployed may be at elevated risk of RA, relative to being deployed without combat experience [33]. This observed association may have been mediated by PTSD, though this was not examined in the previous study. In the present study, it is noteworthy that the observed association between PTSD and risk of any of the selected autoimmune diseases did not differ according to prior combat exposure.

It has been hypothesized that an association between PTSD and autoimmune disease may be at least partially explained by unhealthy behaviors [17, 19, 34, 35]. However, we found that health behaviors—body size, smoking history, and alcohol use—did not appear to influence the association between PTSD and risk of selected autoimmune diseases. Specifically, when these health behaviors were added to the overall model, the magnitude of the effect estimate for PTSD changed by less than 1%. Similar to our findings, previous studies of civilian nurses found that adjustment for smoking had little impact on the association between PTSD and risk of RA [17] and that adjustment for several health behaviors only slightly attenuated the association between PTSD and risk of SLE. Furthermore, there was no indication that smoking mediated the relation between PTSD and autoimmune disorders.

While the underlying biological mechanisms of the relationship between PTSD and autoimmune disorders are not well understood, the concordant findings from this and prior studies suggest that unhealthy behaviors may not strongly impact the relation between PTSD and autoimmune disorders. Rather, the body of evidence suggests that the association may be more likely due to biological changes that occur in the body among those with PTSD, or perhaps even those with high levels of stress. These changes may adversely impact the immune system through increased inflammatory activity, changes in immune-related gene expression, and accelerated senescence of immune cells [10, 12, 13, 15, 16].

While it is known that there are gender effects across a range of autoimmune diseases, with the exception for MS, sex did not significantly moderate the association between PTSD and autoimmune disorders. These results are consistent with previous findings from the VHA study, which reported the magnitude of association between PTSD and each autoimmune disease was similar between men and women [16]. However, based on the increased magnitude of association for MS among men and IBD among women, further exploration of these associations between unique exposures and PTSD-associated immune dysregulation could be necessary.

Notable limitations of this analysis include the lack of medical record review to confirm diagnoses of autoimmune disease identified through electronic medical encounter data. However, the use of administrative data minimizes bias that may occur from reliance on self-reported diagnoses of autoimmune disease. It is possible that the observed associations may reflect differential healthcare utilization by PTSD status where individuals with PTSD may be more likely to seek care and thus more likely to be diagnosed with autoimmune disorders. However, we conducted additional analyses using self-reports to assess the outcomes and our findings showed a similar, yet even stronger association between PTSD and development of self-reported autoimmune disease (results not shown in tables). PTSD was assessed using the PCL-C based on the DSM-IV criteria and thus may reflect general distress; however, this approach has been corroborated by previous studies that observed both high sensitivity and specificity [36]. While the DSM-IV was used because data collection pre-dated the publication of the DSM-5th edition (DSM-V), future studies should replicate these findings using DSM-V criteria. Combat was assessed from a measure with only 5 items; however, a recent study found that this combat measure performed similarly to a more detailed measure [37]. While the relatively short average length of follow-up reduced statistical power for examining potential effect modification and estimating relative risks specific to the rarer autoimmune diseases of interest, this limitation could be mitigated in future research incorporating extended follow-up of this cohort.

Despite these limitations, one of the key strengths of this analysis was the large representative sample of military service members. The prospective design of the study allowed for assessment of PTSD prior to diagnosis of autoimmune disease. Furthermore, cases of autoimmune disease were ascertained from a military healthcare system accessible to all active duty service members. Due to oversampling, the study population included a large proportion of women who have disproportionately higher rates of many autoimmune diseases, and we were able to examine possible gender differences in the associations of interest. We were able to examine the association according to specific types of traumatic experiences that are relatively common in a military population, and we also had information to assess the influence of health behaviors on observed associations.

## Conclusions

Our findings from this analysis contribute to an emerging body of evidence that suggests that PTSD may be a risk factor for autoimmune disease. Furthermore, we observed that the magnitude of this association appears to be similar among those with and without a history of specific traumatic experiences such as combat exposure and physical or sexual trauma. The present study suggests that further research is needed to understand the underlying biological mechanisms which may link PTSD with increased risk of immune-mediated inflammatory conditions, as well as how severity of PTSD may link with increased autoimmune disease risk or severity of illness, and understand whether successful treatment of PTSD may mitigate the risks of these non-neuropsychiatric complications.

## Data Availability

The datasets analyzed during the current study are not publicly available due institutional regulations protecting service member survey responses but are available from the corresponding author on reasonable request (may require data use agreements to be developed).

## Abbreviations

BMI: Body mass index
CI: Confidence interval
DMDC: Defense Manpower Data Center
DSM-IV-TR: Diagnostic and Statistical Manual of Mental Disorders 4th edition
HR: Hazard ratio
IBD: Inflammatory bowel disease
ICD-9-CM: International Classification of Diseases, 9th Revision, Clinical Modification
MDR: Military Health System Data Repository
MS: multiple sclerosis
NHRC: Naval Health Research Center
PCL-C: PTSD Checklist−Civilian Version
PHQ: Patient Health Questionnaire
PTSD: posttraumatic stress disorder
RA: Rheumatoid arthritis
SLE: Systemic lupus erythematosus
US: United States
VHA: Veterans Health Administration
